# Increased Brain Glutathione Levels by Intranasal Insulin Administration

**DOI:** 10.3390/cimb47040284

**Published:** 2025-04-17

**Authors:** Taisuke Kawashima, Wattanaporn Bhadhprasit, Nobuko Matsumura, Chisato Kinoshita, Koji Aoyama

**Affiliations:** Department of Pharmacology, Teikyo University School of Medicine, 2-11-1 Kaga, Itabashi, Tokyo 173-8605, Japan; tamako144@gmail.com (T.K.); sumidaw@med.teikyo-u.ac.jp (W.B.); nmatsumu@med.teikyo-u.ac.jp (N.M.); ciitaka@med.teikyo-u.ac.jp (C.K.)

**Keywords:** glutathione, insulin, EAAC1, GTRAP3-18, neurodegeneration

## Abstract

**Background:** This paper investigates the effect of intranasal insulin administration on brain glutathione (GSH) levels and elucidates the potential mechanism by which insulin enhances antioxidant defenses in the brain. **Methods:** C57BL/6J mice were intranasally administered insulin (2 IU/day) or saline for 7 days. GSH levels were measured in the brain and liver. Blood glucose concentrations and daily food intake were also monitored. Protein levels of excitatory amino acid carrier-1 (EAAC1), its interaction with glutamate transport-associated protein 3-18(GTRAP3-18), and activated AMP-activated protein kinase (AMPK) were assessed. **Results:** Insulin-treated mice exhibited significantly higher GSH levels in the hippocampus and midbrain compared to saline-treated controls, while no significant differences were found in liver GSH levels, blood glucose concentrations, or food intake. EAAC1 expression increased in both the cytosolic and plasma membrane fractions of insulin-treated mouse brains. Furthermore, the interaction between EAAC1 and its negative regulator, GTRAP3-18, along with activated AMPK levels, was reduced in insulin-treated mice. **Conclusions:** Intranasal insulin administration enhances brain GSH levels through a mechanism involving EAAC1 upregulation and reduced AMPK activation. These findings suggest that intranasal insulin could be a promising strategy for enhancing antioxidant defenses against neurodegeneration in the brain.

## 1. Introduction

Glutathione (GSH) is a significant neuroprotective compound [1]. It is a tripeptide composed of cysteine, glutamate, and glycine, and it functions as an antioxidant in the brain to counteract oxidative stress. Neurons are particularly susceptible to oxidative stress, which can lead to neurodegeneration in the brain. This vulnerability arises from the high levels of unsaturated fatty acids in the brain, which generate oxidative stress, and relatively low GSH levels in neurons compared to glial cells. This disparity amplifies the impact of oxidative stress on neurons.

Elevated oxidative stress disrupts the redox balance in the brain, precipitating it towards a pro-oxidant state and impairing normal cellular functions. This imbalance can lead to neurodegeneration. Protecting against GSH depletion in the brain can help safeguard against neurodegeneration caused by oxidative stress.

It is a common observation that GSH depletion occurs in the brains of patients with neurodegenerative diseases such as Alzheimer’s disease (AD), Parkinson’s disease, and amyotrophic lateral sclerosis [1]. However, the treatment of patients with neurodegenerative diseases faces challenges due to the presence of the blood–brain barrier (BBB). The BBB restricts the entry of drugs administered peripherally or GSH itself into the brain, limiting the effectiveness of potential therapies.

In recent years, the “Nose-to-Brain” pathway has gained significant attention as a non-invasive means of delivering drugs directly to the brain [2]. Even for transporting high-molecular-weight drugs with brain-targeting properties, intranasal administration has emerged as a promising method that circumvents the BBB. This approach has led to a range of studies exploring the intranasal administration of high-molecular-weight substances [3] for delivery to the central nervous system (CNS). This approach has suggested the potential therapeutic application in certain neurological disorders [4].

Since insulin, a polypeptide hormone secreted by pancreatic β cells to regulate blood glucose levels, was identified in the CNS [5], research has been conducted to elucidate its function within the brain. A body of accumulated evidence indicates that insulin signaling plays pivotal roles in neuronal survival, plasticity, oxidative stress, and neuroinflammation in the brain [6]. Additionally, it has been established that reduced insulin signaling within the brain is a characteristic feature of AD [7].

A systematic review of clinical trials involving intranasal insulin administration has suggested potential cognitive benefits associated with this treatment [8]. Furthermore, a meta-analysis evaluating the safety and efficacy of intranasal insulin in AD patients has demonstrated clinically favorable results [4]. Nevertheless, the precise mechanism underlying the neuroprotective effects of insulin in the brain remains unclear.

In this study, intranasal insulin administration to mice increased GSH levels in the hippocampus and midbrain without affecting those in the liver or blood glucose levels. Insulin increased expressions of excitatory amino acid carrier-1 (EAAC1) on both the plasma membrane and cytosol fractions in the hippocampus, while both phosphorylated AMP-activated protein kinase (AMPK)α (Thr172) levels, which indicate AMPK activation [9], and the interaction between EAAC1 and glutamate transporter-associated protein 3-18 (GTRAP3-18), which holds EAAC1 at the endoplasmic reticulum (ER) in neurons, decreased in the insulin-treated compared to those in saline-treated mouse brains. These results indicate that insulin-induced EAAC1 translocation from the ER to the plasma membrane leads to an increase in neuronal GSH levels in the brain. This study presents a promising strategy involving intranasal administration, which bypasses the BBB, to deliver insulin into the CNS. This approach holds the potential to induce a neuroprotective effect by elevating neuronal GSH levels in the brain.

## 2. Materials and Methods

### 2.1. Animals

Two-month-old male C57BL/6J mice were used for experiments. All mice were housed in a temperature-controlled room at 24 °C under a 12 h light/dark cycle with food and water available ad libitum. The animal experiment protocols were approved by the Teikyo University Animal Ethics Committee (Approval Number: 17-023, Approval Date: 15 February 2018).

### 2.2. Intranasal Administration

Mice intranasally received a 10 μL drop of insulin (1 IU/10 μL, Eli Lilly, Kobe, Japan) dissolved in saline or the same volume of saline to each nostril once a day for 7 consecutive days. These injections were performed under general anesthesia with isoflurane (Pfizer, New York, NY, USA). The blood glucose levels were also measured at the time of administration and 2 h post-administration. Blood samples were collected from the tail vein under mild isoflurane anesthesia at each time point to minimize animal distress. Anesthesia was carefully maintained at a level sufficient to prevent responses to painful stimuli during blood collection. Food intake was also measured and averaged for each treated mouse, which were housed separately and received a diet based on CRF-1 (Charles River Laboratories, Wilmington, MA, USA).

### 2.3. Tissue Sample Preparations

Mice were sacrificed after deep anesthetization with isoflurane and perfused with cold saline for tissue sample preparations. For an experiment at fasted conditions, mice were fasted or unfasted overnight before collecting the brain samples. The samples of the brain and liver were immediately frozen at −80 °C after measuring the tissue weights.

### 2.4. Total GSH Assay

For the GSH assay, the samples of the hippocampus, midbrain, cerebral cortex, hypothalamus, cerebellum, and liver were homogenized with 5% sulfosalicylic acid solution on ice. Total GSH [reduced GSH plus glutathione disulfide] levels were measured by the NADPH-dependent GSH reductase method. Each brain sample was homogenized in a 20-fold volume of 5% sulfosalicylic acid solution on ice to precipitate cellular macromolecules and extract GSH. After centrifugation at 1200× *g* for 15 min at 4 °C, the supernatants were used for the measurement. The solutions were diluted with phosphate-buffered saline containing 1 mM EDTA and adjusted the pH to 7.2–7.4. A reaction mixture containing 10 mM EDTA/3 mM DTNB/4 mM NADPH/2 IU/mL GSH reductase was added to the same volume of diluted brain solution and GSH standard solutions. Total absorbance at 405 nm was measured and calibrated to GSH standards using a DTX 800 Multimode Detector (Beckman Coulter, Brea, CA, USA).

### 2.5. Western Blot

Brain samples were homogenized with RIPA buffer (50 mM Tris-HCl, pH 7.4, 150 mM NaCl, 1% NP-40, 0.25% sodium deoxycholate, 1 mM EDTA, 1 mM PMSF, 1 mM NaF, 1 mM Na_3_VO_4_, and 5 mg/mL each of leupeptin, pepstatin, and aprotinin) on ice. After centrifugation at 15,000 rpm for 15 min, the supernatants were used as samples for Western blot analysis. Fractionated subcellular proteins were extracted with the use of a Plasma Membrane Protein Extraction Kit (BioVision, Mountain View, CA, USA), according to the manufacturer’s instructions. Rabbit anti-EAAC1 (Alpha Diagnostic Int., San Antonio, TX, USA), rabbit anti-Arl6ip5 (Abnova, Taipei, Taiwan), mouse anti-beta actin (Thermo Fisher Scientific, Waltham, MA, USA), rabbit anti-AMPKα (Cell Signaling Technology, Danvers, MA, USA), rabbit anti-phospho-AMPKα (Thr172), and Na^+^-K^+^ ATPaseα1 (Abcam, Cambridge, UK) antibodies were used for the experiments. Bound antibodies were visualized with Amersham ECL Prime Western Blotting Detection Reagent (Cytiva, Marlborough, MA, USA), followed by a LuminoGraph I system (Atto, Tokyo, Japan), which can measure the optical densities of the bands automatically evaluated by ATTO CS Analyzer 4 software (ver. 2.2.3; ATTO, Tokyo, Japan). The band densities of EAAC1 and phospho-AMPKα (Thr172) were normalized in each case to the densities of the beta-actin or AMPK bands from the same sample. The normalized band densities were then expressed as a percentage of control values running on the same membrane.

### 2.6. Immunoprecipitation

Extracts from brain tissues were preincubated with 75 µL of protein G Plus/Protein A agarose (Merck Millipore, Billerica, MA, USA) to remove nonspecific binding proteins. After centrifugation at 1200× *g* for 15 min, the supernatant was incubated with rabbit anti-Arl6ip5 (GTRAP3-18) antibody (Abnova, Taipei, Taiwan) overnight at 4 °C, followed by incubation with 75 µL of protein G Plus/Protein A agarose for 3 h at 4 °C. The pellets were collected by centrifugation and washed 3 times with RIPA buffer. The samples were then boiled for 3 min to dissociate the immune complexes and centrifuged again at 1200× *g* for 15 min, after which the supernatant was used for Western blot analysis of both EAAC1 and GTRAP3-18.

### 2.7. Statistical Analysis

Data are expressed as means ± SEM. Statistical significance was determined using man Whitney’s *U*-test for two-group comparisons. *p* < 0.05 was considered to be significant.

## 3. Results

**Figure 1:**
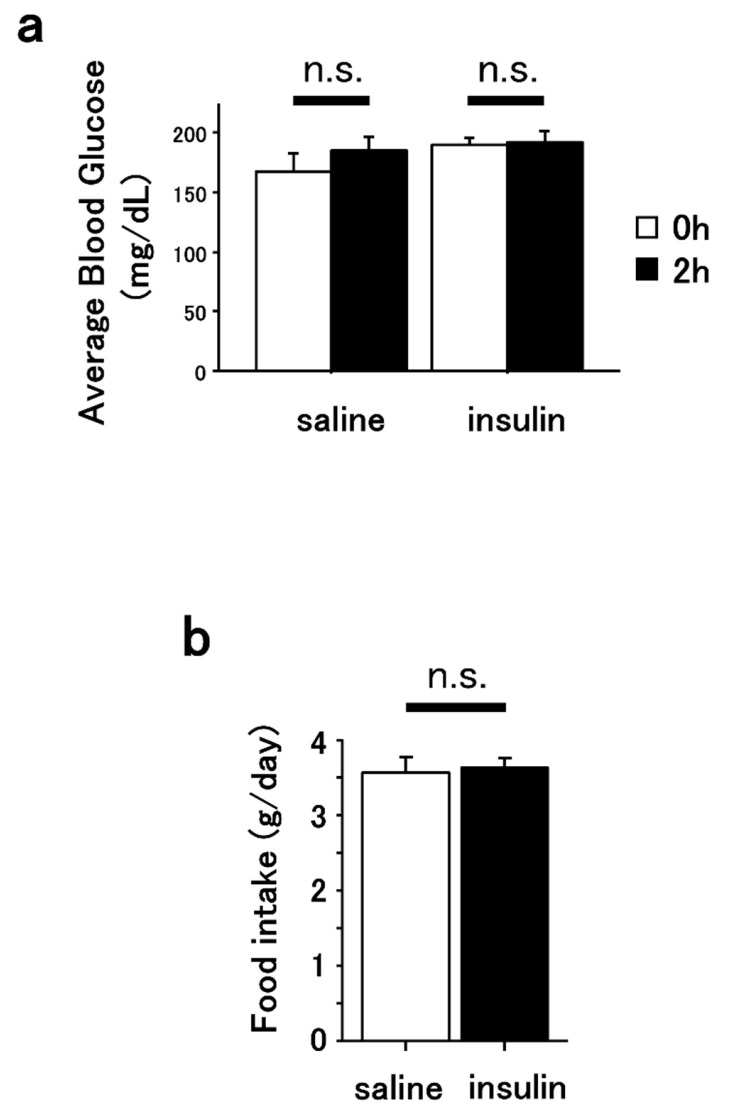
Effect of intranasal insulin administration on blood glucose and food intake. (**a**) Averaged blood glucose levels at 0 and 2 h after intranasal saline and insulin administration in mice are shown. *n* = 3. (**b**) Daily food intake in mice treated with intranasal saline or insulin is shown. *n* = 6. n.s. indicates not significant.

Blood glucose levels were measured at the time of administration and 2 h post-administration with no significant differences in both saline and insulin groups (Figure 1a). The insulin-treated mice did not show any hypoglycemia-induced abnormal behavior, such as generalized seizures or impaired consciousness, throughout the 7-day period following the intranasal injections. Also, the total amounts of daily food intake were not significantly different between saline and insulin-treated mice (Figure 1b).

**Figure 2:**
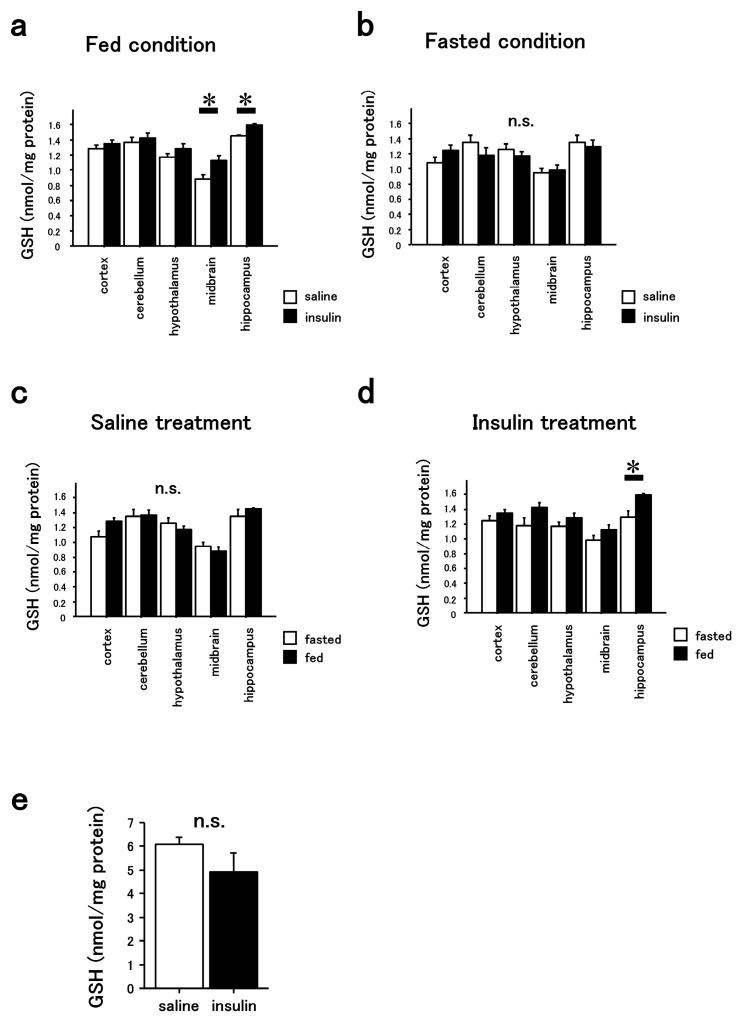
Increased levels of GSH in the hippocampus and midbrain by intranasal insulin administration in mice under fed conditions. (**a**) GSH contents were increased in the hippocampus (*n* = 6) and midbrain (*n* = 3) of insulin-treated mice compared to saline-treated mice under fed conditions. * *p* < 0.05. Other brain regions (cortex; *n* = 9, cerebellum; *n* = 9, hypothalamus; *n* = 6) showed comparable values. (**b**) None of the brain regions (cortex, cerebellum, hypothalamus, midbrain, hippocampus; *n* = 3 for each) of insulin-treated mice showed an increase in GSH levels under fasted conditions. Data from both (**a**,**b**) were also analyzed by both saline (**c**) and insulin (**d**) treatments. GSH levels were elevated in the hippocampus of insulin-treated mice under fed conditions, but not under fasted conditions. * *p* < 0.05. (**e**) GSH levels in the liver of insulin-treated and saline-treated mice under fed conditions were comparable. *n* = 3. n.s. indicates not significant.

GSH levels were significantly higher in both the hippocampus and midbrain of insulin-treated mice compared to those of saline-treated mice under fed but not fasted conditions (Figure 2a,b). Insulin but not saline treatment increased GSH levels in the hippocampus under fed conditions compared to fasted conditions (Figure 2c,d). In the other parts of the brain or the liver, GSH levels were not significantly different between the groups, even under fed conditions (Figure 2a,e).

**Figure 3:**
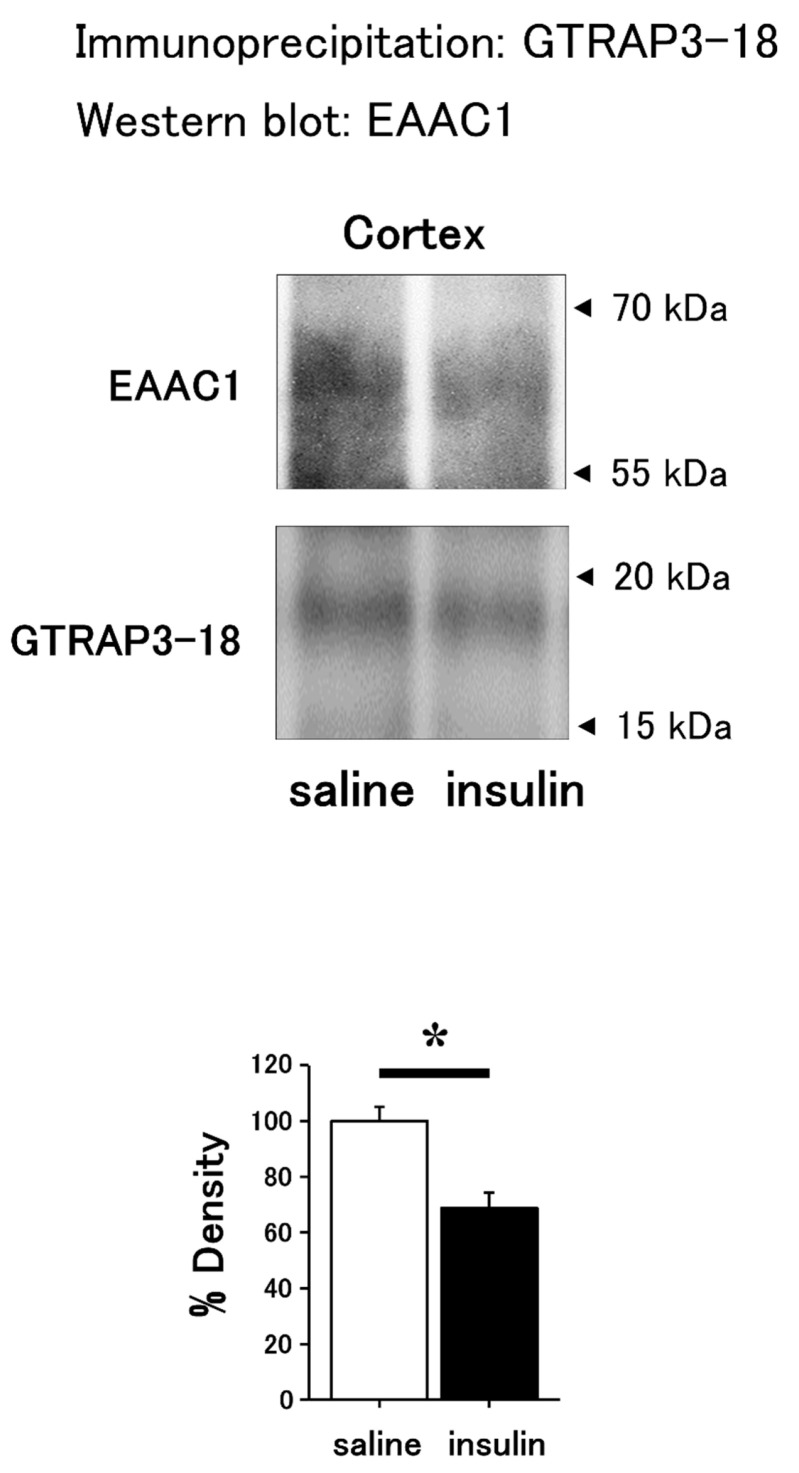
Decreased interaction between EAAC1 and GTRAP3-18 in the mouse brain following intranasal insulin administration. Immunoprecipitation using anti-GTRAP3-18 antibodies and Western blot analysis using anti-EAAC1 and anti-GTRAP3-18 antibodies were performed on the brains of mice treated with intranasal insulin compared to saline. Quantification of protein interaction (upper panel data) by densitometry is shown in the lower panel. *n* = 3. * *p* < 0.05.

**Figure 4:**
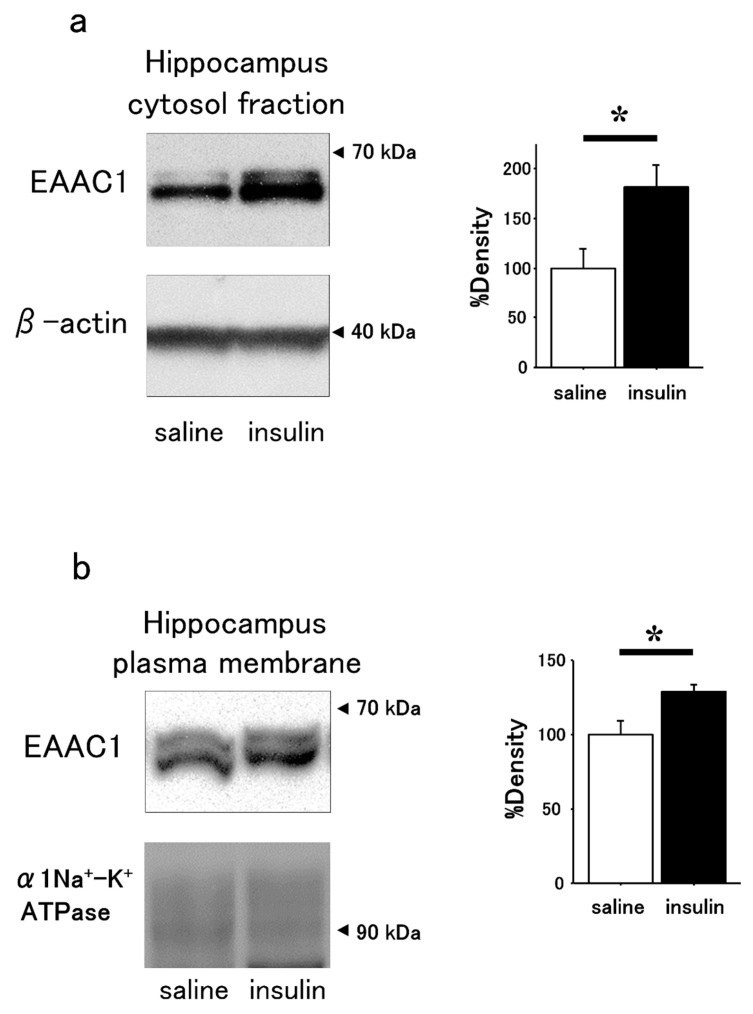
Increased EAAC1 protein expression in the hippocampal cytosol and plasma membrane of intranasal insulin-treated mice. (**a**) Western blot analysis of EAAC1 in the hippocampal cytosol fraction of intranasal insulin-treated mice compared to saline-treated mice. Quantification of EAAC1 protein expression (left panel data) by densitometry is shown in the right panel. *n* = 3. * *p* < 0.05. (**b**) Western blot analysis of EAAC1 levels in the hippocampal plasma membrane of intranasal insulin-treated mice compared to saline-treated mice. Densitometric quantification of EAAC1 protein expression (left panel data) is shown in the right panel. *n* = 3. * *p* < 0.05.

The band densities of EAAC1 in Western blots using brain samples after immunoprecipitation by GTRAP3-18 antibody were significantly decreased in the insulin-treated compared to saline-treated mice (Figure 3). Western blots using samples of the hippocampus showed increased band densities of EAAC1 in the cytosol (Figure 4a) and membrane fractions (Figure 4b) in insulin-treated compared to saline-treated mice.

**Figure 5:**
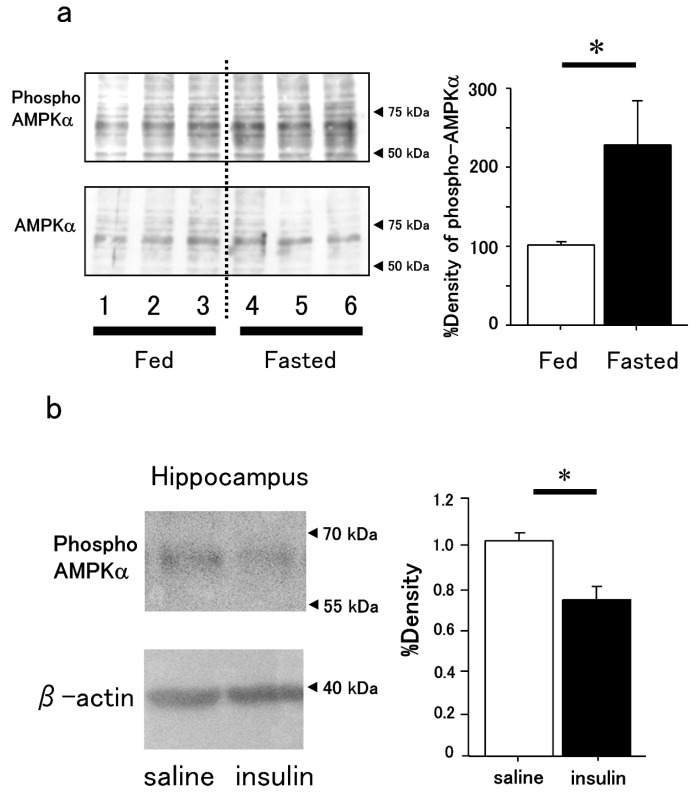
Effect of food intake on phosphorylated AMPKα in the mouse brain and decreased phosphorylated AMPKα protein expression in the hippocampus of intranasal insulin-treated mice. (**a**) Increased phosphorylation of AMPKα in the brain of fasted mice compared to fed mice. Protein expression levels of phosphorylated AMPKα and AMPKα in the brains of fed and fasted mice are shown. Quantification of phosphorylated AMPKα (left panel data) by densitometry is shown as the ratio of phosphorylated AMPKα to total AMPKα protein expression. *n* = 3. * *p* < 0.05. (**b**) Western blot analysis of phosphorylated AMPKα levels in the hippocampus of intranasal insulin-treated mice compared to saline-treated mice. Densitometric quantification of phosphorylated AMPKα protein expression (left panel data) is shown in the right panel. *n* = 4. * *p* < 0.05.

In Figure 5a, the results of Western blots show the increased phosphorylated AMPKα bands of brain samples extracted under fasted conditions compared to those under fed conditions without any treatment. The band densities of phosphorylated AMPKα were decreased in the hippocampus of insulin-treated mice compared to those of saline-treated mice (Figure 5b).

## 4. Discussion

Through intranasal administration, drugs can be directly transported to the brain without being absorbed into the bloodstream [10]. Insulin, in particular, can be transferred to the CNS, including the hippocampus, through intranasal administration, influencing related signaling pathways [11]. While the precise proportion of insulin reaching the brain was not measured in this study, previous research has demonstrated successful CNS delivery of insulin via the intranasal route [12,13,14]. The conditions used here were based on concentrations previously shown to elicit neuroprotective effects in earlier studies. As a limitation, brain glucose concentrations were not assessed. Future studies should address this to better elucidate the relationship between insulin action and central glucose dynamics.

In this study, the intranasal administration of insulin did not significantly decrease blood glucose levels in treated mice, even though the insulin dosage used would typically induce hypoglycemia when administered subcutaneously or intraperitoneally to mice. Importantly, GSH levels were significantly elevated in both the hippocampus and midbrain following intranasal insulin administration, while no such increase was observed in the liver. These findings indicate that the effects of insulin, administered intranasally, are localized to the brain without affecting the peripheral tissues. Although the current GSH assays focused on selected brain regions that were technically feasible to sample in sufficient quantities for analysis, future studies may benefit from including dorsal pontine structures. These regions could provide insights into serotonergic involvement, which may play a role in the central regulation of insulin actions in the brain.

This study shows that the intranasal administration of insulin increases GSH levels in both the midbrain and hippocampus under fed conditions (Figure 2a). These results indicate that insulin may influence antioxidant capacity in specific brain regions. In contrast, under fasted conditions, insulin administration did not lead to significant changes in GSH levels in either region (Figure 2b), suggesting that the metabolic state affects the brain’s response to insulin.

When comparing the effects of insulin between fed and fasted states, a significant increase in hippocampal GSH levels was observed in insulin-treated fed mice, whereas no such difference was found in saline-treated mice (Figure 2c,d). This suggests that the hippocampus may respond differently to insulin depending on nutritional status. These findings imply that insulin’s impact on redox regulation in the brain is both region- and state-dependent.

Insulin is also known to regulate appetite through the hypothalamus [15]. The activation of insulin signaling in the hypothalamus typically leads to reduced appetite through altered expressions of anorexigenic and orexigenic peptides [15]. The insulin doses administered in this study did not influence appetite or hypothalamic GSH levels in the treated mice. These findings indicate that intranasal administration is an effective approach for delivering insulin to the CNS, resulting in increased GSH levels in the hippocampus and midbrain, without affecting the hypothalamus, at least with the insulin dosages employed in this study.

There is a report indicating that GSH levels in the rat cerebral hemisphere and cerebellum decrease during fasting compared to the fed state [16]. Maintaining physiological levels of GSH requires a supply of dietary amino acids, and the observed reduction in brain GSH levels during fasting suggests that GSH synthesis is suppressed under such conditions. Since the intranasal administration of insulin increases GSH levels in the fed state but not in the fasted state, it is possible that insulin exerts a stimulatory effect on the GSH synthetic pathway.

Insulin is not exclusively produced by β cells in the pancreas but also by GABAergic neurons and choroid plexus epithelial cells in the brain [17]. Insulin receptors (IRs) are widely distributed throughout the CNS, including regions such as the hippocampus and midbrain [15]. The stimulation of IRs by insulin triggers IR autophosphorylation in neurons, leading to the activation of cAMP-inhibiting G protein Gi signaling via Gαi2 [18], which has been associated with neuroprotection in the brain [19]. Prior research has suggested that aberrant insulin signaling can contribute to neurodegeneration, and as a result, intranasal insulin administration is regarded as a promising strategy for treating AD [4]. Nevertheless, the precise mechanisms responsible for the neuroprotective effects remain unclear.

EAAC1 is widely expressed throughout the CNS, including in the hippocampus and midbrain [20], and involved in neuronal cysteine uptake rather than in glutamate clearance in the brain [21]. Indeed, cysteine transport is the rate-limiting step for GSH synthesis in mature neurons [1]. Although EAAC1 is mainly localized in the intracellular compartment, once stimulated, EAAC1 is translocated from intracellular compartments to the cell surface [22]. This translocation is inhibited by AMPK activation [23] or GTRAP3-18 [24]. In this study, fasted mice showed AMPK activation and diminished the effect of insulin on GSH levels in the brain. The fed state may amplify insulin’s antioxidant effects via enhanced sensitivity at the cellular level. This could modulate AMPK activity and upregulate EAAC1, thus facilitating increased GSH synthesis in specific brain regions.

Remarkably, the decreased interaction between EAAC1 and GTRAP3-18 with increased EAAC1 in the cytosolic and plasma membrane fractions by the treatment of intranasal insulin suggests that insulin-induced signaling regulates brain GSH levels by controlling the interaction between EAAC1 and GTRAP3-18. A study supporting our findings has also demonstrated that AMPK activation downregulates the expression of EAAC1 on the plasma membrane [23]. IRs coupled to the inhibitory G-protein Gi inhibit the cAMP-dependent signaling pathway in response [19]. Considering the stimulatory regulation of AMPK by cAMP signaling [9], the inhibitory effect of insulin on cAMP-dependent AMPK activity would promote EAAC1 function to increase neuronal GSH levels in the brain. In this study, the AMPKα (Thr172) phosphorylation was inhibited in the brains of insulin-treated mice. The findings of increased EAAC1 expressions on the plasma membrane with increased GSH levels are also supported by our previous studies [25], which have shown a mechanism of EAAC1/GTRAP3-18-mediated signaling that regulates neuronal GSH synthesis in the brain. Indeed, EAAC1-deficient mice showed age-dependent brain atrophy both with learning/memory dysfunction and decreased GSH levels in the brain [26]. Recent clinical studies using proton magnetic resonance spectroscopy revealed that brain GSH levels were decreased in patients with some neurodegenerative diseases compared to healthy subjects [27,28]. Promoting the function of EAAC1 to increase neuronal GSH levels in the brain would be a promising strategy for the treatment of neurodegenerative diseases.

The oral and intravenous administration of insulin are not viable for clinical use due to issues of poor absorption and hypoglycemia, respectively. In contrast, intranasal insulin administration has the potential to modulate neuronal insulin signaling in the brain without impacting systemic glucose metabolism [29]. Our data indeed demonstrated that insulin-induced EAAC1 translocation to the plasma membrane resulted in increased GSH levels in both the hippocampus and midbrain without affecting blood glucose levels or appetite. This study not only elucidates the mechanism by which insulin increases GSH levels in the brain but also suggests a plausible strategy for the clinical application of intranasal insulin administration in the treatment of neurodegenerative diseases.

## Data Availability

The authors confirm that the data supporting the findings of this study are available within the article.
